# Role of Oxidative Stress in Hepatic and Extrahepatic Dysfunctions during Nonalcoholic Fatty Liver Disease (NAFLD)

**DOI:** 10.1155/2020/1617805

**Published:** 2020-10-19

**Authors:** Andrea Gonzalez, Camila Huerta-Salgado, Josué Orozco-Aguilar, Francisco Aguirre, Franco Tacchi, Felipe Simon, Claudio Cabello-Verrugio

**Affiliations:** ^1^Laboratory of Muscle Pathology, Fragility and Aging, Department of Biological Sciences, Faculty of Life Sciences, Universidad Andres Bello, Santiago, Chile; ^2^Millennium Institute on Immunology and Immunotherapy, Santiago, Chile; ^3^Center for the Development of Nanoscience and Nanotechnology (CEDENNA), Universidad de Santiago de Chile, Santiago, Chile; ^4^Millennium Nucleus of Ion Channels-Associated Diseases (MiNICAD), Universidad de Chile, Chile; ^5^Laboratory of Integrative Physiopathology, Department of Biological Sciences, Faculty of Life Sciences, Universidad Andres Bello, Santiago, Chile

## Abstract

Nonalcoholic fatty liver disease (NAFLD) is a pathology that contains a broad liver dysfunctions spectrum. These alterations span from noninflammatory isolated steatosis until nonalcoholic steatohepatitis (NASH), a more aggressive form of the disease characterized by steatosis, inflammatory status, and varying liver degrees fibrosis. NAFLD is the most prevalent chronic liver disease worldwide. The causes of NAFLD are diverse and include genetic and environmental factors. The presence of NASH is strongly associated with cirrhosis development and hepatocellular carcinoma, two conditions that require liver transplantation. The liver alterations during NAFLD are well described. Interestingly, this pathological condition also affects other critical tissues and organs, such as skeletal muscle and even the cardiovascular, renal, and nervous systems. Oxidative stress (OS) is a harmful state present in several chronic diseases, such as NAFLD. The purpose of this review is to describe hepatic and extrahepatic dysfunctions in NAFLD. We will also review the influence of OS on the physiopathological events that affect the critical function of the liver and peripheral tissues.

## 1. Introduction

Chronic liver disease (CLD) is a group of pathologies with abnormal liver function caused by genetic and/or ambient factors [1, 2]. The most common causes of CLD are hepatitis viral infection (hepatitis B and C), alcohol abuse (alcoholic liver disease), nonalcoholic fatty liver disease (NAFLD), autoimmune hepatitis, primary biliary cirrhosis, and primary sclerosing cholangitis. CLD can progress to cirrhosis and then lead to hepatic failure and/or hepatocellular carcinoma (HCC), conditions that may need liver transplantation (LT) [1–4].

NAFLD corresponds to the entire spectrum of fatty liver diseases, characterized by ≥5% hepatic fat accumulation (steatosis in the liver), discarding that this fat accumulation is secondary to significant alcohol consumption, the use of steatogenic medication, or hereditary [5]. NAFLD includes a wide variety of liver disorders ranging from fatty liver to nonalcoholic steatohepatitis (NASH) [3].

In recent years, significant advances have been made in describing NAFLD status, indicating that this is a pathology that affects not only the liver but also other organs and tissues, such as skeletal muscle and the cardiovascular, renal, and nervous systems. A central factor involved in the physiopathology of NAFLD is oxidative stress (OS).

The purpose of this review is to describe the hepatic and extrahepatic dysfunctions in NAFLD and the influence of OS on the pathological mechanisms and events observed in each tissue.

## 2. Nonalcoholic Fatty Liver Disease: A General Overview

NAFLD has a high prevalence because it depends on the population's lifestyle and risk factors, such as obesity, type 2 diabetes mellitus, metabolic syndrome, and hypertriglyceridemia [3, 5–7]. The prevalence of NAFLD corresponds to a 25% worldwide; its prevalence in North America is 24%; South America, 32%; Europe, 20% to 30%; Asia, 15% to 40%; Africa, 13.48%; Middle East, 31.79%; Australia, 40%; and New Zealand, 13% [7, 8].

There are intrahepatic alterations in NAFLD, such as matrix deposits, inflammation damage, parenchymal cell death, angiogenesis, fibrosis, and fat deposits accumulation. These alterations also affect the liver's ability to regenerate and increase portal pressure (portal hypertension) [2, 3].

But NAFLD also affects other tissues than the liver. Thus, the principal intra- and extrahepatic complications associated with the advanced stage of NAFLD are sarcopenia, cirrhotic cardiomyopathy (CCM), portal hypertension, hepatorenal syndrome, hepatic encephalopathy (HE), and peripheral neuropathy (PN). Other dysfunctions are ascites, gastroesophageal varices, liver cancer, coagulopathy, spontaneous bacterial peritonitis, malnutrition, metabolic and immune system abnormalities, and fragility [9–13].

A key element in intra- and extrahepatic disorders in NAFLD is OS produced by an increase in oxidative species and a decrease in antioxidant systems [14, 15]. In the liver, the increase in free fatty acids (FFA) and lipid overload is critical components that can increase reactive oxygen species (ROS) and decrease the antioxidant system in NAFLD. The main mechanisms behind OS are related to cellular organelles' malfunction, such as mitochondrial dysfunction and endoplasmic reticulum (ER) stress, damaging the liver structure and hepatic function. In turn, these structural and functional alterations of liver tissue by ROS influence the detrimental effects in extrahepatic tissues and organs, such as skeletal muscle, the heart, blood vessels, the kidney, and the peripheral and central nervous systems.

## 3. Influence of Oxidative Stress in Nonalcoholic Fatty Liver Disease

### 3.1. Oxidative Stress: General Features

In the organism, cells regularly and continuously produce reactive species, such as ROS and reactive nitrogen species (RNS). ROS and RNS are closely related to each other; so typically, the term ROS is used to represent both oxidant species [16]. ROS is fundamental for the normal function of the immune response, metabolism, and cellular proliferation and differentiation at a low amount. ROS act as signaling molecules to modulate several cell functions in diverse cell types [16, 17]. ROS production involves enzymatic and nonenzymatic reactions in the cytoplasm, cell membrane, ER, mitochondria, and peroxisome or through enzymes such as nicotinamide adenine dinucleotide phosphate (NADPH) oxidase (NOX), xanthine oxidase (XO), cytochrome P450 2E1, cyclooxygenases, and lipoxygenases [16, 18].

ROS are classified as free radicals and nonradical species. Among free radicals are superoxide anion (O_2_·^−^), hydroxyl radical, nitric oxide (NO)-, nitrogen dioxide radical, carbonate radical anion, and alkoxyl/alkyl peroxyl. Among the main nonradical species are hydrogen peroxide, hypochlorous acid, and peroxynitrite/peroxynitrous acid [16, 19, 20].

A balance between ROS and antioxidant systems is critical to ensure proper cell function [16, 20, 21]. There are enzymatic and nonenzymatic antioxidant systems. Among the enzymatic systems are superoxide dismutase 1-3 (SOD), catalase (CAT), glutathione peroxidase 1-8 (GPX), and peroxiredoxin 1-6. Among the nonenzymatic molecules are glutathione (GSH), thioredoxin (TRX), uric acid, vitamins E and C, and bilirubin [16]. In normal conditions, antioxidant systems avoid cellular damage by neutralizing oxidative species [22, 23].

When an imbalance between oxidant species and antioxidant systems occurs in favor of oxidants, it is called OS. This imbalance leads to a disturbance in signaling and redox control and/or damage in various cell structures, such as organelles, proteins, lipids, and membranes, affecting their functions and contributing to the physiopathology of multiple chronic conditions, including NAFLD [16–20].

In the next sections, we will detail the main conditions that can produce OS in the liver and extrahepatic tissues during NAFLD.

### 3.2. Oxidative Stress in the Liver during Nonalcoholic Fatty Liver Disease

**Figure 1:**
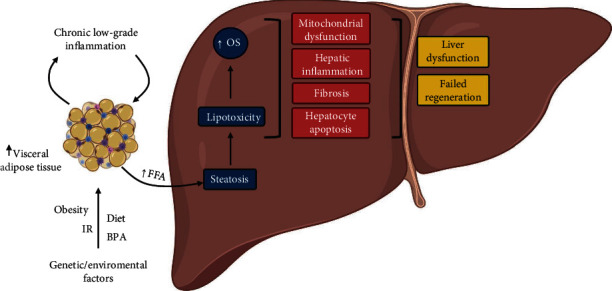
Intrahepatic complications in nonalcoholic fatty liver disease (NAFLD). NAFLD's pathophysiology is affected by genetic and environmental factors such as diet, bisphenol A (BPA), leading to obesity and insulin resistance (IR). Adipose tissue gain contributes to chronic low-grade inflammation and increases free fatty acids (FFA) mobilization, resulting in visceral and ectopic fat deposition. In NAFLD, one of the main alterations is the hepatic steatosis. Thus, steatosis increases FFA, which increases intrahepatic triglycerides levels. This significant lipidic increase inside the liver results in lipotoxicity and oxidative stress (OS). OS and lipotoxicity induce mitochondrial dysfunction, hepatocyte apoptosis, and hepatic inflammation, increasing profibrotic factors that contribute to liver fibrosis. Besides, there is a failed attempt to regenerate the liver. Together, these tissue alterations contribute to hepatic dysfunction. Together, the impaired lipidic metabolism, the increase of proinflammatory cytokines, and the OS can induce hepatic dysfunction that favors NAFLD progression. Created with http://BioRender.com.

In NAFLD, the hepatic functions are altered, and the liver develops steatosis, injury, hepatocyte ballooning, the formation of Mallory–Denk bodies (Mallory hyaline), inflammation, fibrosis, and cell death by apoptosis [13]. NAFLD's pathogenesis involves multiple factors contributing to its development, as indicated in multiple-hit theory [14, 15, 24]. This theory considers that environmental influences can induce weight gain, increased FFA mobilization, fat deposition, and insulin resistance (IR), facilitating lipolysis and the development of chronic low-grade inflammation. These changes increase the flux of FFA to the liver and hepatic lipogenesis de novo, producing hepatic steatosis (see Figure 1) [15, 25, 26]. In the liver, triglycerides and FFA induce lipotoxicity (LTX) and OS, leading to hepatic inflammation, mitochondrial dysfunction, hepatocyte apoptosis, and fibrosis (see Figure 1) [15, 25, 26]. The progressive death of hepatocytes because of increased OS promotes cirrhosis and HCC [26].

Environmental factors may also play a role in the pathogenesis of NAFLD. In this sense, it has been identified that endocrine disruptors, such as bisphenol A (BPA), interfere with physiological hormonal signaling, increasing the risk of developing NAFLD. BPA is a food contaminant widely used to make polycarbonate and epoxy resin plastics. In the liver, exposure to BPA induces epigenetic changes capable of determining triglycerides accumulation through a multifactorial alteration that includes IR, increased *de novo* lipogenesis, and unbalanced lipid homeostasis favoring the NAFLD pathogenesis (see Figure 1) [27–29].

Besides, a consequence of liver dysfunction by NAFLD, which has adverse effects on extrahepatic tissues, is increased ammonia in the blood. Hyperammonemia (HA) is due to the liver's deficiency in converting ammonia to glutamine and urea, accumulated in the blood [30].

In summary, in NAFLD, several factors are involved in the disease's pathogenesis, and they contribute to the development of intrahepatic complications. Among these factors are hepatic steatosis, inflammation, hepatocyte apoptosis, and fibrosis. These complications are responsible for worsening the disease's outcome and can be associated with extrahepatic consequences, which will be reviewed in this section.

#### 3.2.1. Organelles as Sources of Oxidative Stress

One of the OS sources is mitochondria. The normal function of mitochondria includes the regulation of oxidative metabolism, a critical event involved in maintaining the energy balance and normal function of hepatocytes. Among the processes modulated by the mitochondria are *β*-oxidation, the tricarboxylic acid cycle, ketogenesis, electron transport chain (ETC) activity, and adenosine triphosphate (ATP) synthesis [17, 31].

During NAFLD, there is a dysfunction in *β*-oxidation that induces a decreased hepatic peroxisome proliferator-activated receptor alpha (PPAR*α*) activity, increasing liver lipids, modulating lipid homeostasis and inflammation, and favoring the progression of this pathology [32]. Regarding OS, several alterations in mitochondrial function affect ROS production during the progress of NAFLD. Thus, a high ROS production is generated by the deterioration of the ETC, causing an *electron leakage* that leads to an enhanced reaction between electrons and oxygen. This reaction produces ROS and dissipates membrane potential, decreasing the ATP synthesis [17, 31, 33, 34]. The ROS excess oxides phospholipids, such as cardiolipin, a specific phospholipid of the internal mitochondrial membrane. This oxidation promotes the decrease of ETC activity, the opening of the mitochondrial permeability transition pore (mPTP), and the release of cytochrome c to the cytosol, inducing the apoptotic pathway dependent on the caspases [33, 35, 36].

During the early stages of NAFLD, there is an increase in mitochondrial activity as a compensatory mechanism to protect hepatic cells against the harmful effects of lipid storing [36]. This mitochondrial adaptation to avoid excess FFA in hepatocytes increases the production and accumulation of ROS. Thus, the oxidant environment increases the activation of proinflammatory and apoptotic signal pathways [33, 35]. Moreover, mitochondrial activity is regulated by external factors such as BPA, which determines an overload of metabolic pathways in liver cells leading to their *β*-oxidation in the mitochondria and the formation of ROS and mitochondrial dysfunction due to the consumption of NADP^+^ [37].

Smooth and rough ER are abundant in the liver. ER is a fundamental organelle in calcium homeostasis and for the synthesis, folding, and traffic of proteins [17].

Under conditions of LTX and high flow of FFA, such as occurs in NAFLD, unfolded and misfolded proteins are accumulated, leading to a process known as unfolded protein response (UPR), which generates stress in the ER that increases ROS production [38].

Another factor involved in the ER-induced ROS production during NAFLD is intracellular calcium flux. The intracellular calcium flux can decrease the Sarco/ER Ca^2+^-ATPase (SERCA) activity and the ER's calcium-retaining protein by NAFLD-induced FFA increment. This excess calcium released into the cytoplasm is absorbed by the mitochondria, inducing the opening of the mPTP and favoring ROS increase [17, 39, 40].

**Figure 2:**
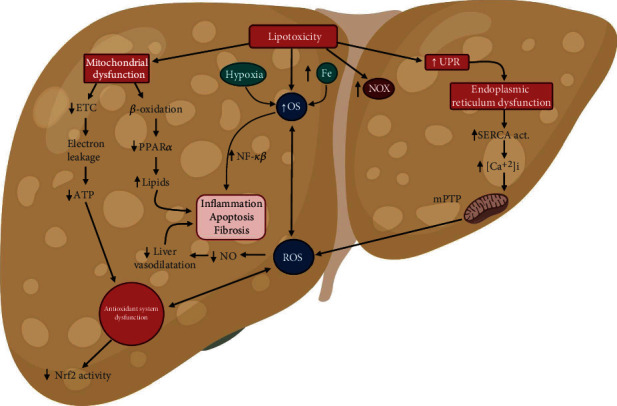
The effect of oxidative stress (OS) in hepatic tissue during nonalcoholic fatty liver disease (NAFLD). In NAFLD, significant lipidic increase inside the liver results in lipotoxicity, which induces oxidative stress (OS), with a marked reactive oxygen species (ROS) increase. OS is the main contributor to NAFLD development due to decreased antioxidant systems, mitochondrial dysfunction, and an increase in unfolded protein response (UPR) by endoplasmic reticulum (ER) stress. Furthermore, an OS increment is due to NAFLD's negative consequences as the iron increase and hypoxia. Lipotoxicity given by NAFLD can directly induce OS and induce organelle damage, as mitochondrial and ER dysfunction. Also, there is an impairment in *β*-oxidation due to a decrease in peroxisome proliferator-activated receptor alpha (PPAR*α*) activity, which increases intrahepatic lipids levels, inducing hepatic inflammation. At the same time, the phospholipid oxidation in the mitochondrial membrane decreases electron transport chain (ETC) that increases electron leakage, diminishing adenosine triphosphate (ATP) production, and generating antioxidant systems dysfunction characterized by the decrease in nuclear factor-(erythroid-derived 2) (Nrf2) activity. Together, these mechanisms related to mitochondrial dysfunction increases OS. On the other hand, lipotoxicity induces an increase of UPR, causing ER dysfunction. The ER dysfunction increases Sarco/endoplasmic reticulum Ca^2+^-ATPase (SERCA) activity and intracellular Ca^+2^ (Ca^+2^i) levels, leading to the opening of the mitochondrial permeability transition pore (mPTP), causing an increase in ROS production. The ROS increment causes a decrease in nitric oxide (NO) levels, causing a reduction in the liver's vasodilatation. Together, all these mechanisms increase ROS production, increasing OS in hepatic tissue, which causes inflammation, hepatocytes apoptosis, and fibrosis during NAFLD progression. Created with http://BioRender.com

In summary, lipid overload and an increase in FFA, typically increased in NAFLD, produces mitochondrial dysfunction and ER stress. These two processes increased ROS production and decreased antioxidant response in liver tissue, which triggers a proapoptotic and inflammatory pathway contributing to NAFLD progression (see Figure 2).

#### 3.2.2. Antioxidant System Dysfunction

In patients with NAFLD, there is a reduction of the antioxidant capacity in hepatic cells [41]. Clinically, the enzymatic (CAT, SOD, and GPX) and nonenzymatic antioxidant systems (GSH, TRX, *α*-tocopherol, and ubiquinone) are decreased in the blood, serum, plasma, and liver [17, 42–44]. The increased ROS in NAFLD can directly deplete antioxidant molecules and inhibit antioxidant enzymes [17].

During NAFLD, the nuclear factor-(erythroid-derived 2) (Nrf2), a transcriptional regulator of antioxidant proteins, is depleted concomitantly to an impaired antioxidant function [41]. In turn, this effect favors the fibrosis, inflammation, and progression of fatty accumulation in the liver [45–47].

In summary, increased ROS production can deplete antioxidant molecules and inhibit antioxidant enzymes' activities, leading to an increase of ROS levels. A key event for the impaired antioxidant response during NAFLD is the decrease in Nrf2 activity (see Figure 2).

#### 3.2.3. Harmful Effects of Oxidative Stress in Liver

Chronic liver injury by NAFLD can progress to parenchymal scarring, cellular dysfunction, and, ultimately, to organ failure, in which OS has a crucial role [48]. The main OS-dependent alterations observed in NAFLD are liver cell abnormalities and endothelial dysfunction (ED).

Hepatic LTX produces OS, which activates Kupffer cells (KC) and hepatic stellate cells (HSC), inducing fibrosis in hepatic tissue [48]. OS-induced KC activation triggers an innate and adaptive immune response with the release of proinflammatory cytokines and chemokines, which activate natural killer T cells and HSC [48, 49]. This response is reinforced by OS generated in hepatocytes. Thus, OS activates the nuclear factor-kappa *β* (NF-*κβ*) pathway, causing the production of proinflammatory cytokines favoring apoptosis of hepatocytes and fibrosis [41, 48].

During NAFLD, there is excess free iron in the liver, accelerating ROS production and OS. Iron overload-induced OS has different effects in hepatic cells: (1) it might directly catalyze lipid peroxidation, resulting in the production of malondialdehyde, which is involved in the fibrogenesis by HSC activation; (2) it reduces antioxidant capacity, decreasing GSH and thus limiting GPX activity; and (3) it inhibits the antioxidant/anti-inflammatory mechanism [34]. Thus, excess iron accumulates in the hepatocytes, leading to OS, which is also associated with inflammation and fibrosis [50].

Patients with NAFLD present endothelial dysfunction (ED), which is the main alteration in the hepatic vascular endothelium. The upregulation of NOX-1 mediates NAFLD-induced ED in the liver. The excess ROS produced by NOX-1 is capable of reducing the NO bioavailability. A consequence of the decreased NO is the decline in vasodilation response and liver circulation and the decrease in its anti-inflammatory, antithrombotic, antifibrogenic, and antioxidant properties in the endothelium [51]. Besides, hypoxia observed from damage in the pericentral region of the liver lobule and around the portal vein could induce OS-dependent hepatic angiogenesis and vascular growth, leading to ED [49].

In summary, hepatic LTX, hypoxia, and iron overload produce OS in NAFLD. Both factors induce the secretion of proinflammatory cytokines and the generation of fibrosis. These responses favor the apoptosis of hepatocytes and, finally, the progression of the pathology. Furthermore, the excess ROS induces ED (see Figure 2).

### 3.3. Adverse Effects of Oxidative Stress in Skeletal Muscle

Sarcopenia is an extrahepatic complication related to skeletal muscle, secondary to NAFLD [52, 53]. Moreover, sarcopenia is characterized by a loss of muscle mass, decreased cross-sectional area of muscle fibers in skeletal muscle. Molecularly, the muscle presents high degradation and/or low synthesis of sarcomeric proteins, mitochondrial dysfunction, autophagy, and OS [54–56].

**Figure 3:**
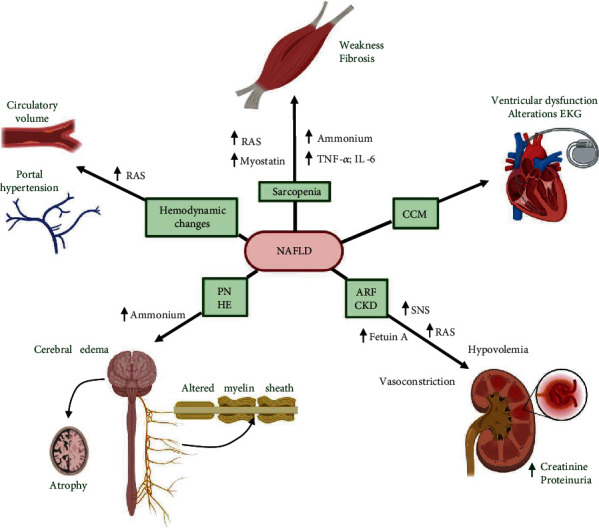
Extrahepatic tissue complications by nonalcoholic fatty liver disease (NAFLD). In skeletal muscle, sarcopenia is induced by NAFLD. The development of sarcopenia is characterized by muscle weakness and fibrosis. Among the factors that contribute to sarcopenia in NAFLD are the increase of proinflammatory cytokines (tumor necrosis factor-alpha (TNF-*α*), interleukin-6 (IL-6)), classical renin-angiotensin system (RAS), myostatin, and ammonium. In cardiac tissue, the main complication induced by NAFLD is cirrhotic cardiomyopathy (CCM). CCM is characterized by systolic and diastolic dysfunction in the ventricle and altered electromechanical patterns. Besides, NAFLD also induces impairment in vascular endothelium, characterized by hemodynamic changes, mainly caused by the increase of classical RAS and represented by the rise in volemia and portal hypertension. NAFLD can induce acute renal failure (ARF) and chronic kidney diseases (CKD) in the renal system. ARF is developed in NAFLD patients with hypovolemia caused by a vasoconstriction response, classical RAS activation, and also activation of the sympathetic nervous system (SNS). CKD is characterized by impaired filtration. This impairment increases creatinine serum levels and induces proteinuria. NAFLD is related to peripheral neuropathy (PN) and hepatic encephalopathy (HE) in the nervous system. PN related to NAFLD is a pathology characterized by altered myelin sheath in peripheral nerves with neuronal loss. The HE causes brain edema and atrophy and is favored by hyperammonemia (HA) in NAFLD. Created with http://BioRender.com

Among the mechanisms involved in the NAFLD-induced sarcopenia is the increased proinflammatory status (by secretion of tumor necrosis factor-alpha (TNF-*α*), interleukin-6 (IL-6), and angiotensin-II (Ang-II) as part of renin-angiotensin system (RAS) pathway) and alteration in muscle protein metabolism (by secretion of myostatin and ammonium) (see Figure 3).

In skeletal muscle, OS leads to the development of sarcopenia and muscular fibrosis secondary to NAFLD. Both pathological states imply a decrease in muscular function, which is mainly related to force generation.

The liver failure causes an increase of soluble molecules in the bloodstream, including TNF-*α*, IL-6, and Ang-II. They are associated with an increased ROS production in skeletal muscle and also with the development of sarcopenia. Ang-II increases the expression and activity of NOX. Concomitant with the high NOX activity, there is an increment of ROS production dependent on this enzyme in skeletal muscle [57, 58]. The NOX-dependent ROS production induced by Ang-II produces muscle atrophy and weakness and decreases in sarcomeric proteins [59].

Another of the soluble factors increased in NAFLD is TNF-*α*, which induces a muscular increase in ROS production. TNF-*α* inhibits the mitochondrial ETC, increasing the ROS production [60]. This increase has been related to the regulation of myogenesis and muscle weakness [59]. IL-6 is another cytokine that influences the increase in ROS through an mPTP-dependent mechanism in skeletal muscle [61, 62].

HA is commonly observed in cirrhosis and plays a significant role in the pathogenesis of sarcopenia. One effect induced by HA is the decrease in muscle protein synthesis, probably by the increased phosphorylation of the eukaryotic initiation factor 2, an essential regulator of protein synthesis involved in translation initiation [63]. This effect has been demonstrated to be mediated by myostatin, which also activates the NF-*κ*B-dependent signaling [64] and promotes autophagy, mitochondrial dysfunction, and OS [64, 65]. Besides, HA induces catabolism of muscle protein and OS-mediated lipid damage, exacerbating sarcopenia [65].

Recently, a murine model of nonalcoholic liver disease has been described with liver damage induced through the hepatotoxin administration [66]. This model produces cholestatic liver failure and develops similar features to patients with NAFLD, including sarcopenia [56]. Intracellularly, hepatotoxin-induced sarcopenia is related to increased ROS production in skeletal muscle. Concomitant to the elevated ROS levels, the muscles present an oxidative-dependent modification of proteins, such as carbonylation and 4-hydroxy-nonenal adduct formation. Besides, there is increased protein catabolism in skeletal muscle through the ubiquitin-proteasome system (UPS), specifically by increasing Muscle RING Finger 1 (MuRF-1) and atrogin-1, which contributes to the development of sarcopenia. Furthermore, OS in skeletal muscle leads to increased myonuclear apoptosis, a particular type of nuclei loss in skeletal muscle that favors the sarcopenia and muscle weakness [67]. The use of N-acetyl cysteine, an antioxidant agent, diminished the apoptotic effect in this model of CLD, recovering muscle function, and force generation [68].

Under OS conditions, the skeletal muscle can develop muscle fibrosis, which is defined as an exacerbated extracellular matrix (ECM) production, decreasing muscle functions, such as movement, contraction, and force generation [69]. The accumulation of interstitial fibrous tissue and muscle atrophy is the major histopathological changes in sarcopenia [70]. Functionally, interstitial fibrosis leads to enhanced muscle stiffness, restricts muscle stretching, and affects the contraction with decreased exercise capacity [71]. Moreover, fibrous tissue deposition might interfere with the interactions between satellite cells and surrounding cells and impair muscle regeneration [71].

The skeletal muscle fibrosis can be induced by several soluble factors such as Ang-II and transforming growth factor-beta (TGF-*β*) [69, 72]. Interestingly, Ang-II and TGF-*β* are increased in oxidative and inflammatory conditions, such as liver diseases [73]. Both factors lead to an increase in ECM components, such as collagens I and III and fibronectin. In muscle fibers, TGF-*β* and Ang-II increase ROS production through the NOX, leading to increased collagen III and fibronectin [74–76]. The ECM accumulation is directly related to OS.

Patients with NAFLD can develop IR and obesity, together with sarcopenia and muscle fibrosis, which can have to OS as a common cause [77]. In obesity (a risk factor of NAFLD), there is sarcopenia, fibrosis, and high lipid content in skeletal muscle, a status known as sarcopenic obesity. The muscle function is also affected in sarcopenic obesity, with low capacity to perform exercise and muscle weakness [78]. One of the highlight changes observed during sarcopenic obesity is the activation of inflammatory pathways that are common to muscle and visceral fat [78]. Obesity activates macrophages, mast cells, and T lymphocytes, promoting a low-level inflammation [79]. These changes induce IR and increased muscle catabolism, which finally leads to gain in fat mass and loss of muscle mass [79]. In obesity, there is also an increase in the release of TNF-*α* and TGF-*β*, which can interact with the fibroblasts found in skeletal muscle, promoting muscle fibrosis [80]. Furthermore, the activation of the Smad-dependent signaling pathway causes the increased secretion of ECM components by fibroblasts reinforcing the fibrotic phenotype [81].

**Figure 4:**
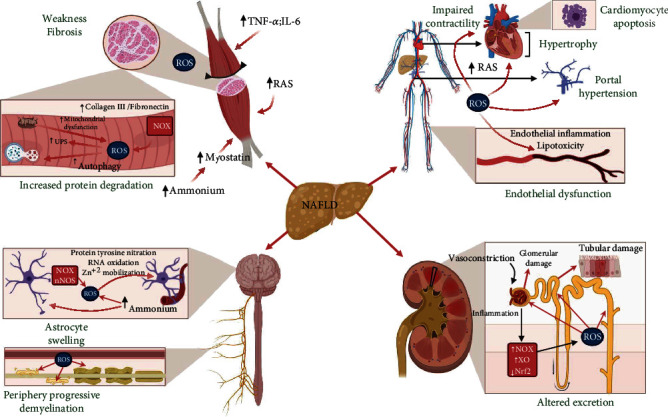
Harmful effects of oxidative stress (OS) in extrahepatic tissues due to nonalcoholic fatty liver disease (NAFLD). In skeletal muscle, NAFLD is responsible for the development of sarcopenia and fibrosis. NAFLD causes an increase of proinflammatory cytokines (tumor necrosis factor-alpha (TNF-*α*), interleukin-6 (IL-6)), reactive oxygen species (ROS) production, classical renin-angiotensin system (RAS) activation, and hyperammonemia (HA), which stimulates an increment in myostatin levels. Together these factors are responsible for increased NADPH oxidase (NOX) activity and mitochondrial dysfunction, which produce ROS. Furthermore, ROS's increment increases the ubiquitin-proteasome system (UPS) and autophagy activity, favoring sarcopenia. Simultaneously, the increase in ROS stimulates higher production of collagen III and fibronectin, contributing to fibrosis in skeletal muscle. Concerning the cardiovascular system, the ROS increment by NAFLD causes impairment in cardiac and vascular functions. ROS is responsible for impaired ion flux, causing impaired contractility, ventricle hypertrophy, and cardiomyocytes apoptosis. ROS and the RAS activity at the vascular level cause portal hypertension and endothelial dysfunction (ED), characterized by endothelial inflammation and lipotoxicity in the tissue. The renal system suffers alterations in excretion due to glomerular and tubular damage by NAFLD. All these damages are caused mainly by vasoconstriction due to NAFLD, which causes hypoxia, inflammation, increasing NOX, xanthine oxidase (XO) activity, and reducing nuclear factor-(erythroid-derived 2) (Nrf2) levels, raising even more ROS levels. At the central nervous system, NAFLD causes hyperammonemia (HA), which provoked astrocyte swelling. Astrocyte swelling increases NOX and nNOS activity, causing an increase in ROS levels. The increment in ROS levels triggers protein tyrosine nitration, ribonucleic acid (RNA) oxidation, and Zn^+2^ mobilization. On the other hand, NAFLD increases ROS levels in the bloodstream; this ROS causes progressive demyelination of peripheral nerves with eventual axonal loss. Created with http://BioRender.com

In summary, patients with NAFLD can develop sarcopenia (muscle weakness) and muscle fibrosis, with OS participation producing a decrease in skeletal muscle function (see Figure 4).

### 3.4. Influence of Oxidative Stress in Cardiovascular Dysfunction

There are several cardiac diseases related to liver disease [82]. The exact mechanism that leads to complications affecting the heart in NAFLD has not been fully described.

The CCM is a condition characterized by the impaired contractile response to stress stimuli, diastolic and systolic dysfunction, electromechanical abnormalities, and autonomic cardiac dysfunction in the absence of other cardiac diseases [83] (see Figure 3).

Besides, the hemodynamic changes are one of the most common alterations in the NAFLD-induced cirrhosis. Among them are hyperdynamic circulation and portal hypertension [83]. The hyperdynamic circulation is a compensatory mechanism to circulatory dysfunction in early cirrhosis stages. This condition activates the RAS and sympathetic nervous system (SNS) to release vasoconstrictor factors to maintain blood pressure [83, 84]. Portal hypertension is a complication present in NAFLD that corresponds to increased vein tension [83] (see Figure 3).

#### 3.4.1. Oxidative Stress Impairs Contractility, Hypertrophy, and Apoptosis in Cardiomyocytes

The main effects of NAFLD in cardiac tissue are related to cardiomyocyte dysfunction. This malfunction involves hypertrophy-dependent alterations in contractility and an increase in the apoptotic rate in cardiomyocytes. These alterations are associated with heart failure in NAFLD [85].

The presence of OS and inflammation produces lower cardiac contractility, as shown in preclinical studies [80, 86]. These pathological events correlate well with the systolic and diastolic dysfunction demonstrated in patients with NAFLD, which produces impaired cardiac contractility [83]. At the cellular level, contractile dysfunction can be explained by a reduced ionic flux, mainly calcium, in cardiomyocytes. This loss of free intracellular calcium homeostasis can be explained by the ROS-dependent impaired function of sarcolemma's L-type calcium channels and also the Na^+^/Ca^2+^ ion exchanger, SERCA2 [85]. Finally, these events induce contractility impairment and an increase in cardiomyocyte apoptosis and cardiac damage in cirrhotic patients [84, 85].

Based on the contractility alteration in NAFLD, the left ventricle induces an adaptive response, leading to hypertrophy [87]. This cardiac hypertrophy is associated with increased ROS levels and activation of the protein kinase C, p38 MAPK, apoptosis-signaling kinase 1, extracellular signal-regulated kinases 1/2 (ERK1/2), Protein kinase B (Akt/PKB), and NF-*κβ* signaling pathways. The activation of these pathways can also increase ROS levels and inflammation and promote OS [85, 86]. The OS-dependent hypertrophy produced in NAFLD involves thickening the ventricular walls, generating arrhythmias, and worsening cardiac diseases, such as CCM [87].

On the other hand, cardiomyocyte apoptosis has been associated with a loss of cardiac tissue function. In this line, the *β*-adrenergic receptor (*β*AR) system regulates cardiomyocyte apoptosis [84, 88]. OS is a critical player in the impaired function of the *β*AR system, leading to cardiomyocyte apoptosis, which directly affects the cardiovascular system in NAFLD [85]. At the molecular level, it has been shown that the OS-induced apoptosis of cardiomyocytes occurs through a mechanism dependent on ERK1/2, c-Jun N-terminal kinase (JNK), p38, and Akt/PKB activation [85].

Therefore, the OS can promote cardiac dysfunction by decreasing the contractility and inducing hypertrophy and apoptosis in NAFLD (see Figure 4).

#### 3.4.2. Oxidative Stress-Dependent Endothelial Dysfunction (ED)

The main effect on blood vessels during NAFLD is ED, which is associated with increased ROS levels. The ROS increment occurs together with NO·– levels and nitric oxide synthase (NOS) uncoupling [89, 90]. NO·– reacts with excessive O_2_·^−^, forming peroxynitrite (ONOO·–), a potent oxidant with cytotoxic activity, that generates vasoconstriction and decreases the NO bioavailability, affecting the vasodilator response [91].

Together, the lower NO synthesis and NOS uncoupling result in the loss of vascular tone regulation, specifically the NO-dependent vasodilatation producing ED. The vascular hypertension is favored by ED, which results in the worsening of the portal hypertension prognosis and contributes to the development of new vascular events, such as atherosclerosis [89] (see Figure 4).

#### 3.4.3. Vascular Endothelial Inflammation Dependent on Oxidative Stress

During NAFLD, the inflammatory state induces cardiokine synthesis and secretion of TGF-*β*1, Ang-II, endothelin-1 (ET-1), and urotensin II, whose increase is related to the development of OS [90, 91].

Ang-II is related to vascular remodeling and ED, which is associated with higher blood pressure and the potent activation of NOX in the endothelium and heart, contributing significantly to ROS production [83, 91]. Ang-II also contributes to ED by its vasoconstrictor activity, generated for decreasing the soluble guanylyl cyclase, leading to an impaired NO·/cyclic guanosine monophosphate signaling. Besides, the increase in Ang-II contributes to vascular endothelium damage dependent on OS, worsening the circulatory system condition in NAFLD [91, 92].

ET-1 is produced in several vascular tissues, including the vascular endothelium. The effects of ET-1 are mediated by their ETA and ETB receptors. ETA mediates the vascular contraction via activation of NOX, XO, lipoxygenase, uncoupled NOS, and mitochondrial enzymes producing OS in the vascular endothelium, promoting inflammatory response, and altering the vascular tone in NAFLD patients [91] (see Figure 4).

#### 3.4.4. Participation of Oxidative Stress in Lipotoxicity (LTX)

In NAFLD, there is an inhibition of very low-density lipoprotein secretion, which induces fatty accumulation [93], a diminution of high-density lipoprotein (HDL) levels, which turn out to be a predictor of lipid peroxidation [89], and the oxidation of low-density lipoprotein (LDL) and HDL. The oxidized LDL (oxLDL) and oxidized HDL are harmful compounds for the organism and are related to atherosclerosis development [89, 94]. Atherosclerosis is the main contributor to coronary artery disease, which has been associated with NASH [87]. In this context, OS-induced oxLDL accumulates in vascular walls and induces leukocyte adhesion and macrophage transformation into foam cells through intracellular fatty accumulation [89, 93]. The mechanisms through oxLDL contribute to the oxidant environment of atherosclerotic injury including the NOX activation, NOS uncoupling, and proinflammatory cytokine production [89]. In endothelial cells, oxLDL can induce inflammasome activation, resulting in decreased cell survival and increased ROS production. oxLDL can inhibit cell proliferation, inhibit cholesterol flux, and induce foam cell apoptosis in the atherosclerotic plaque and vascular endothelium [95]. Together, the increase in ROS and oxLDL not only causes atherosclerosis but is also related to the worsening of the vascular endothelium's condition in NAFLD patients, aggravating their outcome (see Figure 4).

### 3.5. Effects of Oxidative Stress in Renal Alterations

Acute renal failure (ARF) secondary to liver diseases is known as a hepatorenal syndrome, which is potentially reversible and characterized by a reduced glomerular filtration rate and an increase in plasma creatinine, without structural damage in the kidney [96, 97]. Another NAFLD-related disease is chronic kidney disease (CKD), which is defined as renal alterations that produce reduced glomerular filtration and/or the presence of proteinuria with values up to 500 mg [22, 98].

In general, alterations in renal circulation induced by NAFLD are the main mechanisms associated with ARF. Both hypovolemia and RAS/SNS activation produce renal vasoconstriction and alteration in electrolyte secretion, affecting the renal function. Also, lipogenic inflammation and IR can affect renal circulation and promote the secretion of fetuin-A, causing structural damages in renal tissue, leading to CKD (see Figure 3).

#### 3.5.1. Oxidative Stress-Dependent Renal Tissue Damage

As a consequence of liver damage, OS can induce kidney failure, mainly attributed to hemodynamic changes in the kidney, leading to renal ischemia as a feature of NAFLD. In this regard, histological and functional alterations in the kidney were shown in an ischemia-reperfusion model [99]. A similar effect could be observed during renal ischemia caused by liver disease, which could aggravate patients' outcomes related to NAFLD.

Regarding antioxidant systems, Nrf2 activity is essential to protect against OS and inflammation in CKD models and to prevent progression from simple steatosis to NAFLD and cirrhosis. Nrf2 is associated with the expression of a multidrug-resistant protein family of transporters in the kidney and liver. The genetic deletion of the Nrf2 gene affects both organs' function, worsening the NAFLD outcome [45, 100]. In kidney disease, a significant association was observed between low renal Nrf2 activity and high renal dysfunction. These antecedents indicate the importance of antioxidant systems in the development of OS-mediated kidney failure, as found in NAFLD [47, 101, 102].

Finally, the altered function of adipose tissue in NAFLD contributes significantly to RAS activation, which results in an increased Ang-II secretion, promoting renal oxidative damage [103, 104]. The expression of antioxidant systems, such as SOD/CAT or GSH, induces improved kidney function [105]. These results demonstrated the importance of OS in kidney damage, produced by NAFLD (see Figure 4).

#### 3.5.2. Oxidative Stress Alters the Renal Endothelium Function

In liver diseases, there is a severe diminution of renal microvascular flow. This alteration is concomitant with a loss of fenestrations in peritubular endothelial cells and increases in the ammonia levels, as also seen in NAFLD. Besides, there is renal vascular inflammation, which contributes to renal dysfunction [106]. Proinflammatory cytokines upregulate the NOX4 expression in endothelial cells and NOS uncoupling, which results in elevated O_2_·^−^ production. Together, these mechanisms promote renal endothelial injury associated with the proinflammatory state caused by NAFLD [107, 108].

In advanced NAFLD stages, there is a pathological elevation of intra-abdominal pressure, affecting renal blood flow, inducing an increase in blood urea nitrogen (BUN) and serum creatinine. Besides, the elevated renal vascular resistance may result in renal ischemia, which affects the normal excretion process, promoting renal failure associated with NAFLD [109]. Additionally, postischemic periods and renal RAS activation can induce the production of O_2_·^−^ through XO induction and mitochondrial dysfunction, worsening even the OS in renal tissue [110–112]. In renal ischemia, XO activity is associated with oxidative and inflammatory damage. These effects could be correlated with the NAFLD-induced proinflammatory state [113]. Moreover, the renal parenchymal impairment mediated by ischemia due to NAFLD-induced ED is the primary mechanism involved in tubular cell death and interstitial fibrosis in the kidney [96, 106].

The evidence presented in previous sections suggests that hepatic OS during NAFLD could induce renal dysfunction by promoting endothelial and parenchymal injury. The mechanism involved could be the high proinflammatory milieu and intrarenal RAS activation, which are the two critical components mediating the interrelation between liver and renal dysfunctions in NAFLD [99, 113] (see Figure 4).

### 3.6. Effects of Oxidative Stress in Nervous System Dysfunction

There is a close interaction between NAFLD and the development of PN [114]. PN is a disorder in which peripheral nerves are damaged, causing symptoms such as weakness in the extremities, tingling in the hands or feet, and acute pain [115]. From a neurological perspective and based on the damaged neuronal structures, the PNs are classified in axonal, demyelinating, or mixed [116]. Specifically, NAFLD-induced PN is also classified as metabolic neuropathy, characterized by sensorimotor dysfunctions, with a combination of axonal loss, demyelination, and remyelination [116, 117]. It has been reported an essential amelioration of PN after LT showing the influence of liver disease on nervous system dysfunction [118].

On the other hand, HE is a reversible syndrome of cerebral function impairment, manifested in NAFLD's advanced liver complications. HE affects 30%–45% of cirrhotic patients [119]. The symptoms of HE are thinking difficulties, personality changes, low concentration, loss of memory, confusion, anxiety, and reduced visuospatial ability, among others [120].

In nervous tissue, HE induces cerebral atrophy and edema [120, 121]. Besides, HE alters GABAergic, dopaminergic, serotonergic, and glutamatergic neurotransmission [30, 122, 123]. Among the factors contributing to the HE pathogenesis during NAFLD is HA, which affects astrocytes' function and induces brain damage in a ROS dependent mechanism [124] (see Figure 3).

#### 3.6.1. Oxidative Stress-Induced Alterations of Peripheral Nerves

To date, no studies have linked the OS participation in the pathogenesis of PN by NAFLD. [117]. Despite this fact, the damage observed in peripheral nerves during NAFLD-induced PN could be caused by increased ROS, such as reported in other diseases. The contribution of OS in the PN is highlighted. Specifically, OS can alter the lipids present in the myelin sheath that covers peripheral nerves, causing a demyelinating that progressively leads to axonal loss, as demonstrated in diabetic peripheral neuropathy (DPN) [125, 126]. This oxidative damage to the peripheral nerves causes hyperexcitability in the nociceptors, causing neuropathic pain [127]. PN developed in NAFLD or diabetes has typical features such as the increment of ROS levels. Therefore, studies of ROS participation in DPN's pathogenesis could also explain the mechanisms involved in the NAFLD-induced PN (see Figure 4).

#### 3.6.2. Influence of Oxidative Stress on the Central Nervous System

The NAFLD-induced HE is produced due to brain metabolic changes, systemic inflammatory response, and blood-brain barrier alterations [30]. Among the factors involved in the pathogenesis of HE is ammonia [128, 129]. This molecule, considered a neurotoxin, is produced in the gastrointestinal tract [130] and transported via portal circulation to the liver. During NAFLD, the impaired hepatic function affects ammonia metabolism, generating HA conditions in other peripheral tissues, such as brain tissue [30].

The detoxification of cerebral ammonia is mostly carried out in astrocytes through the formation of glutamine from glutamate and ammonia, a process catalyzed by glutamine synthetase [131]. However, in astrocytes, HA causes OS and activation of Na-K-Cl cotransporter (NKCC1), which increases intracellular glutamine levels, causing osmotic astrocyte swelling [132, 133]. Astrocyte swelling, in turn, causes an increase in OS, generating a self-amplification cycle process that contributes to the impaired brain condition observed in HE [132].

The HA-induced OS in astrocytes is generated by a mechanism dependent on the N-methyl D-aspartate receptor (NMDA-R) [134]. Ammonia induces activation of NMDA-R, increasing intracellular Ca^+2^ (Ca^+2^i) levels in astrocytes [135]. High levels of Ca^+2^i trigger NOX and NOS's activation to promote the production of O_2_·^−^ and NO· [133]. These two species can react and form ONOO^−^, which generates OS [132, 136]. The cellular consequences caused by the formation of O_2_·^−^, NO^·^, and ONOO^−^ are ribonucleic acid (RNA) oxidation, zinc mobilization, and protein tyrosine nitration, respectively [124].

RNA oxidation can decrease the efficiency and precision of translation, impairing protein synthesis, and/or generating defective proteins [137]. High levels of oxidized RNA are found in the cerebral cortex of patients with cirrhosis and HE [188]. Among the identified oxidized RNA species in astrocytes during HE is the mRNA encoding the glutamate/aspartate transporter [124], which is involved in the HA-induced OS dependent on the NMDA-R in astrocytes [133, 138–140]. It has also been described that ammonium induces the oxidation of RNA involved in the local protein synthesis in the synapse, a relevant process in synaptic plasticity and memory formation [141, 142]. Interestingly, the oxidation of RNA in the cerebral cortex increases in HE, associated with physical activity impairment [143]. These antecedents suggest that RNA oxidation is a cellular consequence involved in altering brain functions observed in HE.

Zn^+2^ mobilization is another cellular consequence of OS generated in astrocytes in HE. The NO·-dependent S-nitrosylation of metallothionein, a protein that typically sequesters Zn^+2^, causes the release and the increase of intracellular Zn^+2^ concentration [144]. Consequently, there is an activation of the transcriptional factor Sp1 in a Zn^+2^-dependent manner, inducing the gene transcription of the peripheral-type benzodiazepine receptor (PBR) [145]. PBR increases the synthesis of neurosteroids (molecules that have positive GABAergic activity). This increase, in turn, reduced the activity of target neurons [146–148]. Neurosteroids can also bind to the TGR5 receptor expressed in astrocytes and neurons, raising Ca^+2^ and generating ROS, which contributes to the progression of HE [149].

During HE, the tyrosine residue nitration, an OS-induced protein posttranslational modification, affects astrocytes [136]. Exposure of astrocytes to ammonia was identified to cause protein tyrosine nitration in glutamine synthetase and the NKCC1 cotransporter [141, 150]. Nitration of tyrosine residues in glutamine synthase contributes to HA because it decreases enzyme activity dedicated to metabolizing ammonia in astrocytes, favoring ROS production in HE [141, 151]. On the other hand, tyrosine nitration of NKCC1 induces the astrocytic swelling, prooxidant effect, and a self-amplification cycle process that impairs cerebral function in HE [132, 150].

In summary, NAFLD generates an increment in brain ammonia concentration. This HA state is the first stimulus that causes an increase in ROS and RNS in astrocytes, contributing to the pathogenesis of HE. Specifically, oxidative damage triggers RNA oxidation, an increase in intracellular Zn^+2^ concentration, and tyrosine residues' nitration in proteins. All these events converge in the increase in ROS production, feeding back the cycle of oxidative damage to the brain and accelerating the progression of HE (see Figure 4).

## 4. Conclusions

During NAFLD, excessive lipid accumulation occurs in the liver, inducing ROS increment. ROS can cause mitochondrial dysfunction and ER stress in hepatic tissue. Both events increase ROS production. Antioxidant mechanisms are not capable of counteracting the massive ROS increment, which produces OS. Furthermore, OS induces an exacerbated inflammatory response, which triggers apoptosis and fibrosis in hepatic tissue, worsening the liver damage and disease outcome.

The alterations induced by liver damage in NAFLD, such as increased ROS, circulatory cytokines, and HA, are responsible for the injury in peripheral tissues and organs dependent on OS. Thus, in fibers and fibroblasts from skeletal muscle, OS generates sarcopenia and muscular fibrosis. In the heart, the main OS-induced alterations occur in cardiomyocytes, affecting the contractility and heart rate. In the blood vessels, OS induces ED and favors vasoconstriction. In the kidney, OS produces renal failure and endothelial and parenchymal damage through proinflammatory cytokines and RAS activation. Finally, in the nervous system, OS promotes lipid peroxidation in the myelin sheath of peripheral nerves and astrocyte dysfunction.

With the broad spectrum of tissues affected by NAFLD, conducting additional research is essential to establish the mechanisms involved in each complication and thus develop new and specific therapies.

## Abbreviations

Akt/PKB: Protein kinase B
Ang-II: Angiotensin-II
ARF: Acute renal failure
ATP: Adenosine triphosphate
*β*AR: *β*-Adrenergic receptor
BPA: Bisphenol A
BUN: Blood urea nitrogen
Ca^+2^i: Intracellular Ca^+2^
CAT: Catalase
CCM: Cirrhotic cardiomyopathy
CKD: Chronic kidney disease
CLD: Chronic liver disease
DPN: Diabetic peripheral neuropathy
ECM: Extracellular matrix
ED: Endothelial dysfunction
ER: Endoplasmic reticulum
ERK1/2: Extracellular signal-regulated kinases 1/2
ET-1: Endothelin-1
ETC: Electron transport chain
FFA: Free fatty acids
GPX: Glutathione peroxidase
GSH: Glutathione
HA: Hyperammonemia
HCC: Hepatocellular carcinoma
HDL: High-density lipoprotein
HE: Hepatic encephalopathy
HSC: Hepatic stellate cells
IL-6: Interleukin-6
IR: Insulin resistance
KC: Kupffer cells
LDL: Low-density lipoprotein
LT: Liver transplantation
LTX: Lipotoxicity
mPTP: Mitochondrial permeability transition pore
NADPH: Nicotinamide adenine dinucleotide phosphate
NAFLD: Nonalcoholic fatty liver disease
NASH: Nonalcoholic steatohepatitis
NF-*κβ*: Nuclear factor-kappa beta
NMDA-R: N-methyl D-aspartate receptor
NKCC: Na-K-Cl cotransporter
NO: Nitric oxide
NOS: Nitric oxide synthase
NOX: Nicotinamide adenine dinucleotide phosphate oxidase
Nrf2: Nuclear factor-(erythroid-derived 2)
O_2_·^−^: Superoxide anion
ONOO^·–^: Peroxynitrite
OS: Oxidative stress
oxLDL: Oxidized low-density lipoprotein
PBR: Peripheral-type benzodiazepine receptor
PN: Peripheral neuropathy
PPAR*α*: Peroxisome proliferator-activated receptor alpha
RAS: Renin-angiotensin system
RNA: Ribonucleic acid
RNS: Reactive nitrogen species
ROS: Reactive oxygen species
SERCA: Sarco/endoplasmic reticulum Ca^+2^-ATPase
SNS: Sympathetic nervous system
SOD: Superoxide dismutase
TGF-*β*: Transforming growth factor-beta
TNF-*α*: Tumor necrosis factor-alpha
TRX: Thioredoxin
UPR: Unfolded protein response
UPS: Ubiquitin-proteasome system
XO: Xanthine oxidase.
